# Clinical association of body symptoms and primary dysmenorrhea among young and middle-aged women: an observational study

**DOI:** 10.3389/fmed.2025.1529470

**Published:** 2025-03-10

**Authors:** Tzyy-Guey Tseng, Che-Yen Kuan, Yun-Ping Lo, Yun-Shiuan Chuang, Chun-Ying Lee, Yi-Ting Lin, Ing-Luen Shyu, Ming-Tsang Wu, Chi-Jung Tai

**Affiliations:** ^1^Department of Family Medicine, Kaohsiung Medical University Hospital, Kaohsiung Medical University, Kaohsiung, Taiwan; ^2^Department of Obstetrics and Gynecology, Chi Mei Medical Center, Tainan, Taiwan; ^3^Department of Traditional Chinese Medicine, Kaohsiung Medical University Hospital, Kaohsiung Medical University, Kaohsiung, Taiwan; ^4^Research Center for Precision Environmental Medicine, Kaohsiung Medical University, Kaohsiung, Taiwan; ^5^Center for Big Data Research, Kaohsiung Medical University, Kaohsiung, Taiwan; ^6^Faculty of Medicine, College of Medicine, Kaohsiung Medical University, Kaohsiung, Taiwan; ^7^Department of Pharmacy, Chia-Nan University of Pharmacy and Science, Tainan, Taiwan; ^8^Institute of Biomedical Sciences, National Sun Yat-sen University, Kaohsiung, Taiwan; ^9^PhD Program in Environmental and Occupational Medicine, College of Medicine, Kaohsiung Medical University, Kaohsiung, Taiwan; ^10^Department of Family Medicine, Kaohsiung Medical University Gangshan Hospital, Kaohsiung Medical University, Kaohsiung, Taiwan; ^11^Center for Long-Term Care Research, Kaohsiung Medical University, Kaohsiung, Taiwan; ^12^Department of Family Medicine, School of Medicine, College of Medicine, Kaohsiung Medical University, Kaohsiung, Taiwan

**Keywords:** body symptoms, cold extremities, edema, primary dysmenorrhea, risk factors

## Abstract

**Background:**

The mechanism of primary dysmenorrhea remains unraveled. Body symptoms not related to menstrual cycle may indicate the potential mechanism of primary dysmenorrhea, albeit the association has not been proven. Furthermore, we hypothesize that the cumulative burden of these symptoms may influence the incidence of primary dysmenorrhea. Therefore, we aim to design a study to identify bodily symptoms potentially related to primary dysmenorrhea and test the hypothesis in understanding and managing primary dysmenorrhea.

**Methods:**

A total of 3,140 female participants aged 30–50 years were enrolled from the Taiwan Biobank. Stepwise logistic regression was used to select potential body symptoms associated with primary dysmenorrhea from a training dataset. Selected body symptoms were validated in a test dataset. Female participants without dysmenorrhea in the baseline survey were divided into two groups (with and without body symptoms) in a baseline survey. Cox regression and Kaplan-Meier survival analyses were used to evaluate the risk of incident dysmenorrhea.

**Results:**

Women with body symptoms such as cold extremities (adjusted odds ratio [AdjOR], 1.53, 95% confidence interval [CI], 1.12–2.21), dull abdominal pain (AdjOR, 1.45, 95% CI, 1.03–2.04), and edema (AdjOR, 1.43, 95% CI, 1.02–1.99) were significantly associated with dysmenorrhea. Women with the three body symptoms had a significantly higher risk of dysmenorrhea (adjusted hazard ratio, 2.74, 95%CI, 1.18–6.31; log-rank test, *p* = 0.0017) than those without body symptoms. Trend analysis showed that the risk of dysmenorrhea increased with the number of body symptoms (*p*-trend = 0.025).

**Conclusion:**

This study identified cold extremities, dull abdominal pain, and edema as predictors of primary dysmenorrhea, with their accumulation associated with a higher risk of developing dysmenorrhea. We propose that further research explore pharmacological and non-pharmacological interventions targeting these symptoms, as they may provide long-term benefits in the management of primary dysmenorrhea.

## 1 Introduction

Dysmenorrhea is characterized by a painful sensation in the lower abdomen that occurs before and/or during menstruation. Affecting 16%−91% of women during their reproductive age (1), this prevalent gynecological condition poses a significant burden on individuals, healthcare systems, and society at large (2). Notably, dysmenorrhea-related absenteeism and presenteeism among women of reproductive age lead to reduced productivity in both educational and workplace settings, resulting in substantial economic losses. In the United States alone, these losses are estimated at $2 billion annually (3). Consequently, effective management of dysmenorrhea is crucial to mitigating its wide-ranging impacts.

Dysmenorrhea is classified into two types: primary dysmenorrhea, which occurs without an underlying gynecological condition, while secondary dysmenorrhea generally has underlying gynecological disease, such as endometriosis, adenomyosis, or uterine myoma (4). Unlike secondary dysmenorrhea, the pathophysiology of primary dysmenorrhea remains unraveled in modern times. The most widely recognized cause of primary dysmenorrhea is the excessive production of uterine leukotrienes and prostaglandins via the cyclooxygenase (COX) pathway, leading to myometrial contraction and ischemia (5). Despite this knowledge, current treatments targeting prostaglandin and its derivatives are still unsatisfactory, not only because of their possible side effects and allergic reactions. For example, although nonsteroidal anti-inflammatory drugs and hormone therapy are effective in alleviating symptoms for many women (6), they cannot offer a cure or prevent symptom recurrence. Other treatment options aimed at inducing myometrial relaxation, such as glyceryl trinitrate (7), magnesium, and calcium channel blockers remain controversial due to inconsistent clinical evidence (8). Therefore, a variety of nonmedical remedies have been proposed to treat dysmenorrhea. However, the efficacy of herbal remedies (e.g., ginger, rose tea, and sweet fennel seed extract) and dietary supplements (e.g., vitamin E, vitamin B1, and fish oil) for primary dysmenorrhea is supported by limited clinical data (9). This suggests the potential involvement of alternative etiological factors in primary dysmenorrhea.

Mechanoreceptors sensitization is another generally recognized pathophysiological mechanism underlying primary dysmenorrhea (6). To address this, transcutaneous electrical nerve stimulation (TENs) has been employed as a treatment modality, functioning by gating ascending pain signals at the spinal cord level (10, 11). A Cochrane review suggests that both high-frequency and low-frequency TENS can effectively alleviate dysmenorrhea symptoms compared to placebo or no treatment (12). Similarly, acupuncture and acupressure have demonstrated comparable efficacy in providing pain relief for patients with dysmenorrhea (13). Manual therapy and pelvic floor exercises have also been shown to be effective in reducing pain (14).

Early-stage pathophysiological mechanisms may not directly cause primary dysmenorrhea but could lead to mild body symptoms in advance. According to traditional Chinese medicine (TCM) theory, such symptoms may be linked to obstetric and gynecological diseases in women (15). From a TCM perspective, dysmenorrhea is associated with cold accumulation, blood stasis, and Qi stagnation (16), which contribute to composite bodily symptoms and the development of what is termed a TCM body constitution (17). These specific bodily symptoms may serve as risk factors or early indicators of primary dysmenorrhea. To the best of our knowledge, only one previous study reported that lower abdominal pain, which was not related to menstrual periods, was associated with dysmenorrhea with an odds ratio (OR) of 1.80 [95% confidence interval (CI), 1.3–2.3] (18). Identifying bodily symptoms potentially related to primary dysmenorrhea could provide valuable insights for its management. Furthermore, we hypothesize that the cumulative burden of these symptoms may influence the incidence of primary dysmenorrhea. Therefore, we aim to design a study to test this hypothesis and explore the relevance of TCM theory in understanding and managing primary dysmenorrhea.

## 2 Methods

### 2.1 Data source and study sample

The Taiwan Biobank has recruited 139,842 community-based volunteers and 6,834 hospital-based volunteers since 2012 (19). The Taiwan Biobank obtained informed consent from all participants or their legal guardians for research use of the collected data and samples. The current study was approved by the Institutional Review Board-II of Kaohsiung Medical University Chung-Ho Memorial Hospital [approval no. KMUHIRB-E(II)-20230266] in 2023 and all methods were performed in accordance with relevant guidelines and regulations. We adhered to the Strengthening the Reporting of Observational Studies in Epidemiology (STROBE) checklist to ensure comprehensive and transparent reporting of the study findings (20).

In this study, we used a subset of the Taiwan Biobank data by random sampling from 104,451 community-based participants recruited between 2012–2018 (21). The data included basic demographic information, personal healthy behaviors, personal medical history, and body symptoms. Details and regulations of the Taiwan Biobank are described elsewhere (22).

**Figure 1:**
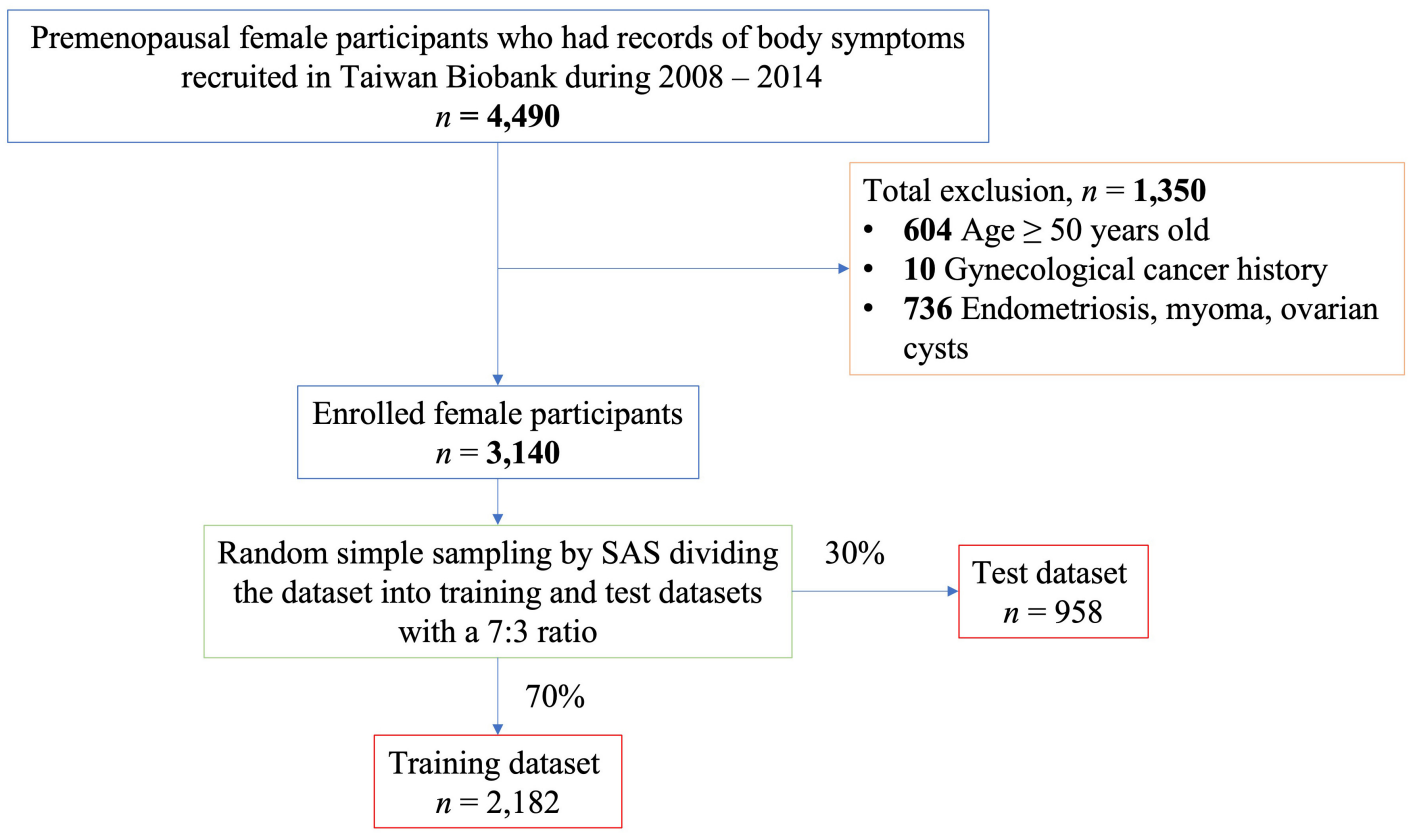
The enrollment process of target female participants and the strategy for selecting training dataset and test dataset.

The unique point of the Taiwan Biobank for women study is that the questionnaire included many questions about associated body symptoms, which were not related to menstrual periods. In the Taiwan Biobank, body symptoms included cold extremities, fatigue, oral ulcers, dull abdominal pain, shortness of breath, chest tightness, tinnitus, ecchymosis, dry skin, varicose veins, soreness (over the waist, knee, or heels), muscle spasm, edema (over the face, eyebrow, or extremities), and hot flushes. However, only a few female participants answered the questions regarding body symptoms. Therefore, 4,490 female participants with records of body symptoms in the Taiwan biobank were selected for further analysis (Figure 1).

### 2.2 Identification of body symptoms associated with primary dysmenorrhea

Targeting at the primary dysmenorrhea, selected female participants went through the exclusion process (Figure 1). First, 604 participants aged ≥50 years were excluded. Second, 10 participants were excluded because of a history of gynecological cancer. Finally, 736 participants with a history of endometriosis, myoma, or ovarian cyst were excluded. After these exclusion, 3,140 female participants were selected using random simple sampling with a 7:3 ratio using SAS (Statistics Analysis System Institute Inc., Cary, NC, USA) software and divided into training (*n* = 2,182) and test (*n* = 958) datasets (Figure 1).

Logistic regression analyses were conducted to identify predictors of primary dysmenorrhea. First, stepwise logistic regression was performed on the training dataset to select the primary body symptoms associated with primary dysmenorrhea. Non-significant body symptoms identified during this process were excluded from further analysis. Finally, the selected body symptoms were validated using multivariable logistic regression on the test dataset.

### 2.3 Cohort study design

**Figure 2:**
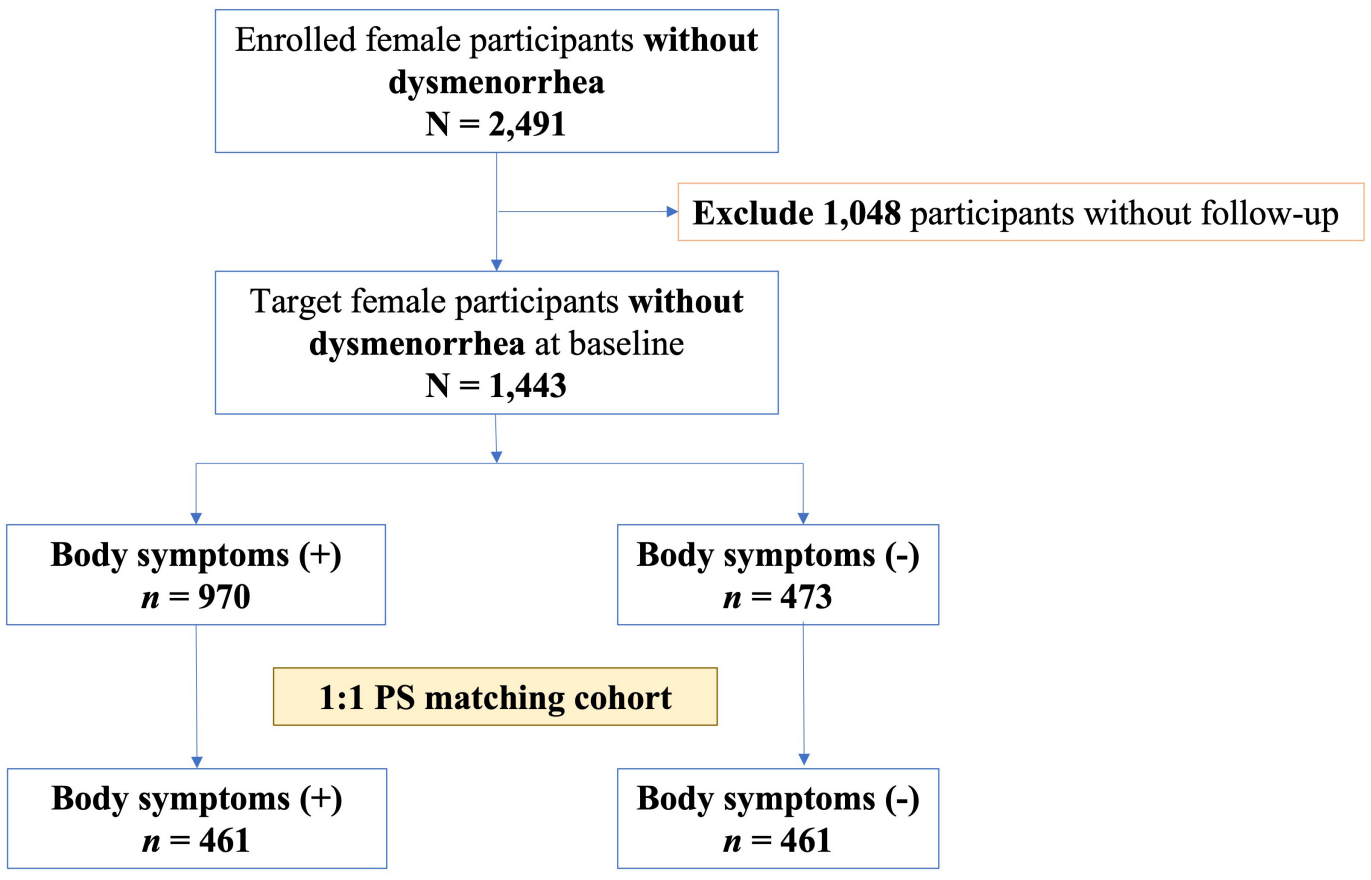
The cohort study flowchart. PS, propensity-score.

To further investigate whether the selected body symptoms led to dysmenorrhea, we designed a retrospective cohort study. Initially, 2,491 female participants without dysmenorrhea in the first wave of the survey were selected. Next, 1,048 female participants were excluded, owing to a lack of follow-up surveys. The participants were then divided into two groups: with and without selected body symptoms (Figure 2). Moreover, we stratified participants in the group with body symptoms into strata, with different numbers of body symptoms in the first wave of the survey.

### 2.4 Covariates

Although direct causation of dysmenorrhea has not been definitively established, previous studies have proposed a conceptual framework that highlights associations between dysmenorrhea and its determinants including lifestyle, reproductive, psychological, and other health-related factors (1, 18). In this study, we adopted this framework to select covariates, which were treated as potential confounding factors in the cohort analysis.

In this study, lifestyle factors included exercise habits, smoking, tea, coffee, and alcohol consumption. Previous studies have shown inconsistent evidence regarding the association between dysmenorrhea and cigarette smoking (5, 23), while alcohol consumption was not associated with dysmenorrhea (24). Unexpectedly, a systematic review and meta-analysis showed that exercise reduced pain intensity in patients with primary dysmenorrhea (25).

Regarding reproductive factors, this study included parity, irregular menstrual cycle, and age at menarche. The association between parity and dysmenorrhea was consistent across previous studies, with reported ORs ranging from 0.2 to 0.74 (1, 5, 24, 26). Irregular menstrual cycle was associated with an increased risk of dysmenorrhea (27). However, the evidence regarding age at menarche as a predictor of dysmenorrhea remains inconclusive. Hu et al. found that an age at menarche ≤ 12 years was a risk factor for dysmenorrhea (OR, 1.16, 95% CI, 1.01–1.33) among Chinese Female University students (28). In contrast, other studies did not observe a higher risk of dysmenorrhea in women with an age at menarche ≤ 12 years (29, 30).

Psychological factors, such as increased levels of stress or depressive mood, were associated with an increased risk of dysmenorrhea (28, 31). This may explain the higher prevalence of dysmenorrhea among adolescent women and those working in healthcare and military services (32, 33). For comorbidities, this study considered personal history of major diseases, including hypertension, asthma, peptic ulcer, diabetes, and low back pain.

For other health factors, body mass index (BMI) was calculated as a person's weight in kilograms divided by the square of their height in meters (kg/m^2^). BMI was categorized as underweight (BMI < 18.5), normal weight (18.5 ≤ BMI < 24.0), overweight (24.0 ≤ BMI < 27.0), or obese (BMI ≥ 27.0), according to the BMI category defined by the Health Promotion Administration, Taiwan (34).

### 2.5 Sensitivity analysis

Propensity score (PS) was calculated using the aforementioned covariates. We then generated a 1:1 PS-matched cohort for sensitivity analysis (35). We used Greedy nearest neighbor matching without replacement. We specified the caliper width of 0.25, which indicated that the difference in PS between the treated unit and its matching control unit must be ≤ 0.25. PS matching were conducted using the PSMATCH procedure provided by SAS. Standardized mean differences were calculated to compare the distribution of baseline covariates between groups with and without body symptoms after PS matching (36). A previous study suggested that a standardized mean difference above 0.1 denoted a meaningful imbalance in the baseline covariates.

### 2.6 Statistical analyses

Descriptive statistics were used to assess patient demographics, number and percentage for categorical variables, and the mean ± standard deviation for continuous variables. Cox regression analysis was performed to estimate the adjusted hazard ratios (adjHRs) and 95% CIs for dysmenorrhea. A trend analysis was also performed to evaluate the accumulating effect of body symptoms on primary dysmenorrhea. We adjusted these models to account for the confounding factors mentioned above. Statistical analyses were conducted using the SAS version 9.4. Using R (version 4.2.0, R Foundation for Statistical Computing, Vienna, Austria) packages (survival, survminer, and dplyr), the Kaplan-Meier survival analysis and log-rank test were used for time-to-event analysis. A *P*-value of < 0.05 was considered statistically significant.

## 3 Results

### 3.1 Body symptoms and primary dysmenorrhea

In general, women with primary dysmenorrhea exhibited a higher prevalence of associated body symptoms (unrelated to menstrual periods), except for varicose veins, compared to those without dysmenorrhea (Table 1). The most prevalent body symptoms in the dysmenorrhea group were fatigue (70.4%), cold extremities (58.4%), and dizziness (58.4%).

**Table 1:** The body symptoms which were not related to menstrual cycles in participants with or without dysmenorrhea (*N* = 3,140).

	**Dysmenorrhea (+) *n =* 649**	**Dysmenorrhea (–) *n =* 2,491**	***P* Value**
**Body symptoms**, ***n*** **(%)**			
Cold extremities	379 (58.4%)	1,162 (46.7%)	< 0.001^*^
Dizziness	378 (58.2%)	1,208 (48.5%)	< 0.001^*^
Fatigue	457 (70.4%)	1,393 (55.9%)	< 0.001^*^
Oral ulcer	202 (31.1%)	648 (26.0%)	0.009^*^
Abdominal dull pain	305 (47.0%)	780 (31.3%)	< 0.001^*^
Shortness of breath	335 (51.6%)	1,043 (41.9%)	< 0.001^*^
Chest tightness	272 (41.9%)	765 (30.7%)	< 0.001^*^
Tinnitus	207 (31.9%)	600 (24.1%)	< 0.001^*^
Ecchymosis	162 (25.0%)	446 (17.9%)	< 0.001^*^
Dry skin	340 (52.4%)	1,104 (44.3%)	< 0.001^*^
Varicose veins	231 (35.6%)	854 (34.3%)	0.53
Waist, knees or heels soreness	372 (57.3%)	1,190 (47.8%)	< 0.001^*^
Muscle spasm	205 (31.6%)	631 (25.3%)	0.001^*^
Edema^§^	289 (44.5%)	750 (30.1%)	< 0.001^*^
Hot flash	133 (20.5%)	396 (15.9%)	0.005^*^

^*^Chi-square test, *p* < 0.05. ^§^ Edema in the face, eyebrow, or extremities.

Using the training dataset, stepwise logistic regression identified cold extremities (AdjOR, 1.38, 95% CI, 1.11–1.71), fatigue (AdjOR, 1.56, 95% CI, 1.22–2.00), dull abdominal pain (AdjOR, 1.61, 95% CI, 1.29–2.02), and edema (AdjOR, 1.56, 95% CI, 1.25–1.96) as major predictors of dysmenorrhea among all associated body symptoms (Table 2). The findings were further validated using multivariable logistic regression on the test dataset, which revealed that only cold extremities (AdjOR, 1.53, 95%CI, 1.12–2.11), dull abdominal pain (AdjOR, 1.45, 95%CI, 1.03–2.04), and edema (AdjOR, 1.45, 95%CI, 1.02–1.99) were significantly associated with an increased risk of dysmenorrhea (Table 2). In the subsequent cohort study, we further investigated whether these selected body symptoms contribute to the incidence of dysmenorrhea.

**Table 2:** Body symptoms in prediction of dysmenorrhea.

	**Training dataset**		**Test dataset**	
**Body symptoms**	**AdjOR (95% CI)**	***P*** **Value**	**AdjOR (95% CI)**	***P*** **Value**
Cold extremities	1.38 (1.11–1.71)	0.004^*^	1.53 (1.12–2.11)	0.009^†^
Fatigue	1.56 (1.22–2.00)	< 0.001^*^	1.17 (0.83–1.66)	0.36
Abdominal dull pain	1.61 (1.29–2.02)	< 0.001^*^	1.45 (1.03–2.04)	0.03^†^
Edema^‡^	1.56 (1.25–1.96)	< 0.001^*^	1.43 (1.02–1.99)	0.04^†^

Odds ratios were adjusted for all body symptoms listed in Table 1. AdjOR, adjusted odds ratio; CI, confidence interval. ^*^Stepwise logistic regression, *p*-value < 0.05. ^†^Multivariable logistic regression, *p*-value < 0.05. ^‡^Edema in the face, eyebrow, or extremities.

### 3.2 Baseline characteristics of the cohort study

Participants without dysmenorrhea (*n* = 1,443) were divided into two groups: 970 (67.2%) participants had at least one of the three selected body symptoms (cold extremities, dull abdominal pain, and edema), while 473 (32.7%) did not suffer from any of these body symptoms (Figure 2). The mean follow-up time was 4.1 ± 1.0 years. Among the group with body symptoms, cold extremities were reported in 68.0% of participants, dull abdominal pain in 44.3%, and edema in 41.4% (Table 3). Over half of the patients (56.4%) reported one body symptom, 33.4% reported two symptoms, and 10.2% reported all three symptoms.

**Table 3:** Baseline characteristics of target female participants before and after 1:1 propensity-score matching.

	**Before matching**		**1:1 PS matching**		
	**Body symptoms (**+**)** ***n** =* **970**	**Body symptoms (–)** ***n** =* **473**	**Body symptoms (**+**)** ***n** =* **461**	**Body symptoms (–)** ***n** =* **461**	**Standardized mean difference**
**Age (year), mean** **±SD**	39.9 ± 5.5	40.8 ± 5.4	40.9 ± 5.4	40.8 ± 5.4	0.013
**Age group**					
30–39	462 (47.6%)	182 (38.5%)	178 (38.6%)	178 (38.6%)	
40–49	508 (52.4%)	291 (61.5%)	283 (61.4%)	283 (61.4%)	< 0.001
**Body symptoms**					
Cold extremities	660 (68.0%)	-	306 (66.4%)	-	
Abdominal dull pain	430 (44.3%)	-	203 (44.0%)	-	
Edema^*^	402 (41.4%)	-	192 (41.7%)	-	
**Number of total body symptoms**					
One	547 (56.4%)	-	265 (57.5%)	-	
Two	324 (33.4%)	-	152 (33.0%)	-	
Three	99 (10.2%)	-	44 (9.5%)	-	
**Obstetric and gynecological history**, ***n*** **(%)**					
Age at menarche, mean ± SD	13.3 ± 1.4	13.5 ± 1.4	13.5 ± 1.5	13.4 ± 1.4	0.006
Irregular menstrual cycle	173 (17.8%)	82 (17.3%)	86 (18.7%)	80 (17.4%)	0.034
Parity	766 (79.0%)	397 (83.9%)	382 (82.9%)	387 (84.0%)	0.028
Hormone contraceptives	33 (3.4%)	11 (2.3%)	12 (2.6%)	11 (2.4%)	0.013
**BMI, kg/m**^2^, ***n*** **(%)**					0.041
Underweight (BMI < 18.5)	51 (5.3%)	15 (3.2%)	16 (3.5%)	15 (3.3%)	
Normal weight (18.5 ≤ BMI < 24.0)	620 (63.9%)	290 (61.3%)	286 (62.0%)	285 (61.8%)	
Overweight (24 ≤ BMI < 27.0)	178 (18.4%)	92 (19.5%)	92 (20.0%)	88 (19.1%)	
Obesity (BMI ≥ 27)	121 (12.5%)	76 (16.1%)	67 (14.5%)	73 (15.8%)	
**Healthy behaviors**, ***n*** **(%)**					
Alcohol	13 (1.3%)	7 (1.5%)	6 (1.3%)	7 (1.5%)	0.017
Smoking	40 (4.1%)	10 (2.1%)	10 (2.2%)	10 (2.2%)	< 0.001
Exercise habits	227 (23.4%)	133 (28.1%)	127 (27.6%)	129 (28.0%)	0.010
Tea	361 (37.2%)	177 (37.4%)	170 (36.9%)	171 (37.1%)	0.004
Coffee	353 (36.4%)	165 (34.9%)	153 (33.2%)	161 (34.9%)	0.036
**Comorbidities**, ***n*** **(%)**					
Hypertension	17 (1.8%)	10 (2.1%)	7 (1.5%)	10 (2.2%)	0.069
Asthma	30 (3.1%)	10 (2.1%)	16 (3.5%)	10 (2.2%)	0.078
Peptic ulcer	111 (11.4%)	41 (8.7%)	48 (10.4%)	40 (8.7%)	0.058
Depression	35 (3.6%)	10 (2.1%)	11 (2.4%)	10 (2.2%)	0.013
Diabetes mellitus	8 (0.8%)	6 (1.3%)	7 (1.5%)	5 (1.1%)	0.035
Low back pain	370 (38.1%)	107 (22.6%)	112 (24.3%)	104 (22.6%)	0.038

^*^Edema in the face, eyebrow, or extremities. BMI, body mass index.

The mean age of participants with and without body symptoms was 39.9 (±5.5) and 40.8 (±5.4) years, respectively. In terms of obstetric and gynecological history, both groups had similar ages at menarche (13.3 ± 1.4 vs. 13.5 ± 1.4 years) and comparable prevalence of irregular menstrual cycles (17.8% vs. 17.3%) (Table 3). However, parity was higher among participants without body symptoms (83.9% vs. 79.0%).

Compared to those with body symptoms, a higher proportion of participants without body symptoms had exercise habits. Additionally, participants with body symptoms had a higher prevalence of peptic ulcers (11.4% vs. 8.7%) and low back pain (38.1% vs. 22.6%) (Table 1).

### 3.3 Outcome of the cohort study

The results showed that the participants with all three body symptoms had a significantly higher risk of incident dysmenorrhea (AdjHR, 2.74, 95% CI, 1.18–6.31) compared to those without any body symptoms (Table 4). While the risk was not statistically significant for participants with one or two symptoms, trend analysis indicated a dose-response relationship, with the risk of dysmenorrhea increasing as the number of body symptoms increased (*p* trend, 0.025).

**Table 4:** Estimates of cox regression investigating associations between the number of body symptoms and dysmenorrhea before and after 1:1 propensity-score matching.

**The number of symptoms**	**Before matching**			**1:1 PS matching**		
	**Adjusted HR (95% CI)**	***P*** **value**	***P*** **for trend**	**Adjusted HR (95% CI)**	***P*** **value**	***P*** **for trend**
0	1.00		0.025^†^	1.00		0.08
1	0.87 (0.42–1.77)	0.69		0.76 (0.29–1.99)	0.58	
2	1.71 (0.86–3.41)	0.13		1.84 (0.77–4.38)	0.17	
3	2.74 (1.18–6.31)	0.019^*^		3.13 (1.03–9.51)	0.048^*^	

CI, confidence interval; HR, hazard ratio; PS, propensity score. Hazard ratios were adjusted for all covariates listed in Table 3. Body symptoms included cold extremities, abdominal dull pain, and edema. ^*^Cox proportional hazard regression, *p* < 0.05; ^†^ *p* for trend < 0.05.

**Figure 3:**
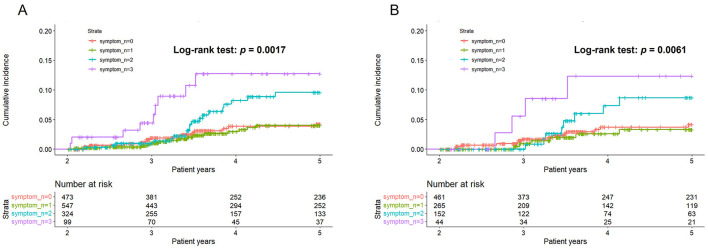
Kaplan-Meier curves of numbers of body symptoms in predicting dysmenorrhea. **(A)** Original cohort. **(B)** Post 1:1 propensity-score matching.

The Kaplan-Meier survival curve demonstrated that the group with three body symptoms had a higher cumulative incidence of dysmenorrhea after two patient-years (Figure 3A). Additionally, the incidence of dysmenorrhea increased among participants with two body symptoms after approximately 3.4 patient-years. The *p*-value for the log-rank test was 0.0017, further supporting these findings.

### 3.4 Sensitivity analysis

After the 1:1 PS matching, 461 participants were included in each group (Table 3). The standard mean difference of all covariates was below 0.1, indicating a well-balanced distribution between the two groups post-matching. Cox regression analysis consistently demonstrated that participants with all three body symptoms had a significant higher risk of incident dysmenorrhea (AdjHR, 3.13, 95% CI, 1.03–9.51) (Table 3). This finding was further supported by Kaplan-Meier survival analysis (log-rank test, *p* = 0.0061) (Figure 3B). Although the risk of dysmenorrhea increased with the number of body symptoms in the PS-matched cohort, the *p*-value for trend did not reach statistical significance.

## 4 Discussion

### 4.1 Main findings and comparison with evidence

Unlike previous studies that primarily focused on adolescents and young women, this study evaluated risk factors for dysmenorrhea in young and middle-aged women. The prevalence of dysmenorrhea in this study was 20.7%, which, while relatively low, was higher than rates reported in studies from Japan (15.8%) (37), Hungary (15.5%) (29), and the United Kingdom (15%) (38). In comparison to a cross-sectional study conducted among adult and middle-aged women in Brazil, the parity rates were similar (80.6% vs. 82.0%) (39). However, the prevalence of irregular menstrual cycles was lower in our study (17.7% vs. 25.5%).

We designed a cross-sectional study and identified three body symptoms predictive of dysmenorrhea in young and middle-aged women: cold extremities (AdjOR, 1.53), dull abdominal pain (AdjOR, 1.45), and edema in the face, eyebrow, or extremities (AdjOR, 1.43) (Table 2). Interestingly, these findings align closely with TCM theory. Cold extremities are indicative of cold accumulation in the body, dull abdominal pain may arise from Qi stagnation in the abdomen, and edema may correlate with blood stasis according to TCM principles.

In Western medicine, it is well known that the presence of multiple abnormal blood test results increases an individual's susceptibility to cardiovascular diseases (40). For example, someone with multiple morbidities, such as impaired fasting glucose, abnormal lipid profiles, and chronic kidney disease, is at a higher risk of ischemic heart disease compared to those with only one abnormality. Through this study, we demonstrated that the accumulation of three specific body symptoms—cold extremities, dull abdominal pain, and edema)—was significantly associated with a higher risk of incident dysmenorrhea, with an AdjHR of 2.74 (Table 4). This finding was further supported by sensitivity analysis using the 1:1 PS-matched cohort.

However, the concept of accumulating specific body symptoms as predictors of diseases is not widely recognized in Western medicine, though it is a common perspective in TCM. Using real-world data, this study is the first to support this perspective in the context of primary dysmenorrhea. Our findings suggest that the combination of three body symptoms may reflect a specific body condition or represent a TCM body constitution, which predisposes female to primary dysmenorrhea. Therefore, this study provides new insights into the potential mechanisms and approaches underlying primary dysmenorrhea.

### 4.2 Strength and limitations

The first strength of this study lies in the unique opportunity provided by the Taiwan Biobank to evaluate the association between body symptoms and dysmenorrhea. Additionally, this study utilized a larger cohort than previous research, comprising over 3,000 participants with detailed records of body symptoms. We also conducted a robust cross-sectional study, using a training dataset to identify associated body symptoms and a test dataset to validate these findings. Furthermore, we demonstrated the association between body symptoms and primary dysmenorrhea through a cohort study.

Moreover, Weissman et al. reported that a 1-year increase in age was associated with an 8% decrease in the risk of developing dysmenorrhea (5). As a result, our findings may represent an underestimation, as the risk associated with accumulated body symptoms could be attenuated by aging.

Although our study generated important findings, these results should be interpreted with caution. First, similar to other observational studies, the current study may be subject to residual confounding factors and cannot establish causality. Second, certain clinical data, such as the severity, duration, and frequency of dysmenorrhea, were not available from the Taiwan Biobank. Additionally, medical records between two waves of survey were absent, preventing us from accounting for the influence of time-dependent covariates. In terms of generalizability, the findings of this study are applicable only to young and middle-aged women and cannot be extended to adolescents. Furthermore, the cohort results were limited to an Asian population and may not apply to Caucasian, Latin, or Black populations.

### 4.3 Potential clinical implications and suggestions for further study

This study proposes a novel approach to dysmenorrhea management by addressing specific body symptoms—such as cold extremities, abdominal dull pain, and edema—rather than solely focusing on pain relief during dysmenorrhea attacks.

From a TCM perspective, cold extremities indicate cold accumulation in the body, while edema may correlate with blood stasis. These symptoms, reflecting poor circulation and overall health, align with Western medicine's understanding of systemic circulatory dysfunction. Notably, peripheral circulation has been linked to conditions like erectile dysfunction in men (41), suggesting a potential connection between peripheral circulation and the circulatory function of internal genital organs in women. Consequently, vasodilators such as Sidenafil citrate (42), a selective phosphodiesterase type-5 inhibitor known to improve genital circulation in men, could be a promising candidate for dysmenorrhea treatment. From another perspective, diuretics, which reduce overall body water volume and alleviate edema, may also decrease the water content within the uterus, potentially relieving dysmenorrhea. However, these hypotheses necessitate further basic research and clinical studies to validate their efficacy.

On the other hand, improving peripheral and general circulation may enhance uterine circulation, potentially preventing, or alleviating primary dysmenorrhea. Previous studies have demonstrated that localized heat application to the suprapubic region provides pain relief and improves quality of life for dysmenorrhea patients (43). Ancient TCM literature highlights the benefits of hot springs for various gynecological conditions, including dysmenorrhea. Similarly, Yang et al. have shown that spa therapy can alleviate general health issues (44). However, as these treatments were studied over relatively short durations, we recommend further research through cohort studies to evaluate their long-term effects on dysmenorrhea prevention and recurrence.

We believe that enhancing general health status and modifying TCM body constitution can contribute positively to dysmenorrhea management. Although previous studies have shown limited evidence for the effectiveness of exercise and yoga in treating dysmenorrhea (45, 46), high-quality cohort studies are needed to clarify their role. In contrast, local manual therapy and pelvic floor exercises have demonstrated beneficial effects on pain relief and reducing pain sensitivity (14). These insights collectively underscore the potential of non-pharmacological approaches in dysmenorrhea management.

## 5 Conclusion

This study identified cold extremities, dull abdominal pain, and edema as predictors of primary dysmenorrhea. The accumulation of these symptoms was associated with an increased risk of incident dysmenorrhea. Both pharmacological and non-pharmacological approaches aimed at alleviating these symptoms may offer long-term benefits in managing primary dysmenorrhea. We believe these findings can pave the way for innovative treatments and help reduce the socio-economic burden associated with primary dysmenorrhea.

## Data Availability

The original contributions presented in the study are included in the article/supplementary material, further inquiries can be directed to the corresponding authors.
